# Machine learning for identifying risk of death in patients with severe fever with thrombocytopenia syndrome

**DOI:** 10.3389/fmicb.2024.1458670

**Published:** 2024-09-13

**Authors:** Qionghan He, Zihao You, Qiuping Dong, Jiale Guo, Zhaoru Zhang

**Affiliations:** ^1^Department of Infectious Diseases, Chaohu Hospital of Anhui Medical University, Hefei, China; ^2^Department of General Medicine, Chaohu Hospital of Anhui Medical University, Hefei, China; ^3^Department of Infectious Diseases, Anhui Public Health Clinical Center, Hefei, China; ^4^Department of Orthopedics, Chaohu Hospital of Anhui Medical University, Hefei, China

**Keywords:** machine learning, severe fever with thrombocytopenia syndrome, public health, risk factors, predictive model

## Abstract

**Background:**

Severe fever with thrombocytopenia syndrome (SFTS) has attracted attention due to the rising incidence and high severity and mortality rates. This study aims to construct a machine learning (ML) model to identify SFTS patients at high risk of death early in hospital admission, and to provide early intensive intervention with a view to reducing the risk of death.

**Methods:**

Data of patients hospitalized for SFTS in two hospitals were collected as training and validation sets, respectively, and six ML methods were used to construct the models using the screened variables as features. The performance of the models was comprehensively evaluated and the best model was selected for interpretation and development of an online web calculator for application.

**Results:**

A total of 483 participants were enrolled in the study and 96 (19.88%) patients died due to SFTS. After a comprehensive evaluation, the XGBoost-based model performs best: the AUC scores for the training and validation sets are 0.962 and 0.997.

**Conclusion:**

Using ML can be a good way to identify high risk individuals in SFTS patients. We can use this model to identify patients at high risk of death early in their admission and manage them intensively at an early stage.

## 1 Introduction

Severe fever with thrombocytopenia syndrome (SFTS) is an emerging infectious disease caused by Dabie bandavirus (DBBV), which was first identified and reported in central and northeastern China in 2011 (Yu et al., 2011), and subsequently reported in South Korea, Japan, Vietnam (Kim et al., 2013; Takahashi et al., 2014; Tran et al., 2019). DBBV belongs to the genus bandavirus of the Phenuiviridae family (Casel et al., 2021), and was first discovered by Chinese scholars in 2009 from ticks (Yu et al., 2011). SFTS is tick-borne and tick-to-human transmission is the main route of SFTS virus infection (Xu et al., 2011). In addition, studies have shown that SFTS virus may be transmitted from person to person through close contact (Tang et al., 2013; Bao et al., 2011). Because of its wide distribution, SFTS has become a major public health risk not only in Chinese but also in other parts of the world (Liu et al., 2014).

The prognostic spectrum of SFTS is broad, with a variety of outcomes ranging from a self-limiting clinical course to life-threatening or even death. Death, as the most devastating clinical outcome, is also the most talked about outcome, and relevant studies have shown that the mortality rates of SFTS are 12–50% (Yu et al., 2011; Li et al., 2018; Gai et al., 2012; Zhang et al., 2012; Yang et al., 2023). The clinical manifestations of SFTS were not specific at admission, and patients with similar symptoms may progress to different prognoses. The analysis of high-risk factors for death in SFTS is still in the exploratory stage at this stage. The aim of this study was to explore the high risk factors for death in SFTS and further construct a risk model for identifying patients at high risk of SFTS at the time of admission. So, it is crucial to identify the risk of death in SFTS patients at an early stage and to intervene in high-risk patients at an early stage.

Machine learning (ML) methods, a subfield of artificial intelligence, is an approach to implementing artificial intelligence that investigates how algorithms can enable computers to learn from data and make predictions or decisions (Marx, 2019), and is divided into categories such as supervised and unsupervised learning (Deo, 2015). ML methods are used in all aspects of modern society (Suwardi et al., 2022; Moriwaki et al., 2023; Bayer and Edwards, 2021), and have shown great potential in many medical fields (Deo et al., 2014). The greatest strength of ML methods is their great performance on many clinically relevant tasks. ML methods can evaluate real-world data, most real-world data are nonlinear, and ML methods can provide more intelligent optimization strategies by learning from historical data and experience to construct models that perform better than traditional linear prediction methods (Deo, 2015).

Given the current widespread application of artificial intelligence in medicine, the use of ML is expected to enable early identification of the risk of death in SFTS patients. The aim of this study is to explore the high-risk factors for death in SFTS patients and to further enable the prediction of the risk of death in SFTS patients based on ML.

## 2 Material and methodology

### 2.1 Data collection

In this study, patients who were discharged from Chaohu Hospital of Anhui Medical University from May 2016 to December 2023 and from Anhui Provincial Public Health Clinical Center (North District of the First Affiliated Hospital of Anhui Medical University) from April 2020 to December 2023 with a final diagnosis of SFTS were collected. Relevant medical records were extracted from the electronic medical record system. In conjunction with previous literature and related research, the general demographic characteristics, common chronic diseases, clinical characteristics and routine laboratory findings were extracted as our extracted variables, specifically: sex, age, days from onset to admission (DFOTA), hypertension, coronary heart disease (CHD), diabetes, cerebral infarction (CI), temperature, pulse rate (PR), respiration rate (RR), systolic blood pressure (SBP), diastolic blood pressure (DBP), myalgia, fatigue, nausea, emesis, diarrhea, abdominal pain (AP), cough, dyspnea, lymphadenopathy, hepatosplenomegaly, disturbance of consciousness (DOC), white blood cell count(WBC), platelet count (PLT), neutrophil count (N), lymphocyte count (L), monocyte count (M), hemoglobin (HB), alanine transaminase (ALT), aspartate transaminase (AST), albumin (ALB), globulin (GLO), potassium ions (K^+^), calcium ions (Ca^+^), glucose (GLU), blood urea nitrogen (BUN), creatinine (CRE), lactate dehydrogenase (LDH), creatine kinase (CK), creatine kinase isoenzyme (CK.MB), prothrombin time (PT), activated partial thromboplastin time (APTT), fibrinogen (FIB), thromboplastin time (TT), D-dimer (D-D). In this case, the laboratory tests were selected from the initial tests performed within 24 h of admission to the hospital. Death was the prognostic indicator studied in this study, and the survival status of the patients at the time of discharge was known through the medical record information in the electronic medical record system, and patients whose survival status at the time of discharge was doubtful were followed up by telephone using the telephone numbers of the patients or their family members retrieved from the electronic medical record system to find out whether they had died after discharge from the hospital.

Inclusion criteria for patients were: patients diagnosed with fever with at the time of discharge from the hospital [the diagnosis met the criteria of the Ministry of Health of the People’s Republic of China’s Guidelines for Prevention and Treatment of Severe Fever with Thrombocytopenia Syndrome (2010 or 2023 edition) (China MoHoPsRo, 2011; China MoHoPsRo, 2024)]. Exclusion criteria for patients were: 1. missing data >20%, 2. unclear prognosis (death), 3. laboratory-confirmed infections with other pathogens such as COVID-19, hantavirus, *Orientia tsutsugamushi*, and rickettsiae, and 4. other major illnesses that severely affected prognosis.

The study was conducted in accordance with the principles of the Declaration of Helsinki. It was approved by the Ethics Review Committee of Chaohu Hospital of Anhui Medical University (Ethics No. KYXM202311006) and the Ethics Review Committee of Anhui Provincial Public Health Clinical Center (Ethics No. PJ-YX2024-027). This was a retrospective study and patients’ personal information was omitted from the analysis. A written informed consent waiver was obtained from the patients based on local policy.

### 2.2 Statistical analysis

First of all, in this study the data is preprocessed. Since the dataset in this study has missing data, we need to explore the features of the missing data and use multiple interpolation to recover the missing data, which is achieved by using the “mice” package (Zhang, 2016; van Buuren and Groothuis-Oudshoorn, 2011). In this study, 500 iterations of 5-fold interpolation technique is used to realize the interpolation of missing data, by comparing the distribution of the original data and the interpolation values, we choose the appropriate interpolation value as the final interpolation value to make the dataset complete.

Next, we performed a one-way analysis of the death and non-death groups of the data from the training set, which was implemented using the “CBCgrps” package (Zhang et al., 2017). Continuous variables were analyzed by the independent samples *t*-test or Mann–Whitney U. Continuous variables with normal distribution were expressed as mean ± standard deviation, and continuous rows of non-normally distributed variables were expressed as median (interquartile range). Categorical variables were analyzed according to distribution using the chi-square test, Wilcoxon rank-sum test, Fisher test, and continuity correction, and were expressed as the number of cases and component ratios. For variables with *p* < 0.1 in the univariate analysis, we included them in the logistic regression model to perform multifactorial regression analysis to evaluate the risk factors for death in SFTS patients. For variables screened by multifactorial logistic regression, we will use restricted cubic spline plots (RCS) to further explore whether there is a nonlinear relationship between them and the outcomes.

### 2.3 Construction and validation of the prediction model

In this study, data from Chaohu Hospital of Anhui Medical University was used as the training set and data from Anhui Public Health Center was used as the external validation set. The training set was used to construct models using different methods and optimized to reduce prediction errors. Then, these models were validated on the validation set to check the robustness of the models. Correlation test is first performed on the screened variables to determine whether there is any multicollinearity among the variables. The correlation coefficient indicates the correlation of one predictor variable with other predictor variables in the data, with absolute values greater than 0.7 indicating strong correlation between the variables, and heat maps were drawn to visualize the results. The filtered variables are incorporated into the machine learning model.

We use six different models of Gradient Boosting Machine (GBM), k-Nearest Neighbors (KNN), Logistic Regression (LR), Neural Network (NNet), Support Vector Machine (SVM), eXtreme Gradient Boosting (XGBoost). 10 times 10 fold cross validation technique is used to avoid comparison bias due to data selection. The area under the ROC curve (AUC), accuracy, recall, specificity, precision, Kappa value, Matthews correlation coefficient (MCC), F1 score, and brier score are used for model discrimination. The Kappa value is used to evaluate the consistency between the predicted and actual values of the model, and the value range is [−1, 1]. Generally believed that *K* > 0.75 means better consistency, *K* between 0.40 and 0.75 means medium and high consistency, *K* < 0.4 means poor consistency, the closer the *K* value is to 1, the better the consistency is. MCC is a balanced metric that not only indicates the correlation coefficient between predicted and true results, but also handles cases where the dataset is unbalanced. It produces high scores only when good results are obtained for all four categories in the confusion matrix: true positives, false negatives, true negatives and false positives. Due to the low incidence of positive events in this study, the MCC value provides a better measure of the accuracy of the multiclassification model under unbalanced distribution compared to other statistical indicators. It takes values in the range of [−1, 1], and the more the value is skewed towards 1, the better the prediction is. Greater than 0.7 indicates high accuracy (Baldi et al., 2000; Chicco and Jurman, 2020). F1 Score is the harmonic mean between precision and recall, which is used for evaluating the accuracy and robustness of the model. Its value ranges from [0, 1], the closer the value is to 1 the better the model accuracy is. Brier score is used to evaluate the calibration of the model, its value ranges from [0, 1], the closer the value is to 0 the better the model calibration is, brier score from 0.1 to 0.25 indicates good calibration, brier score <0.1 indicates excellent calibration. Decision curve analysis (DCA) was used to evaluate the clinical utility of the models (Vickers et al., 2008). Various evaluation metrics were considered together to select the model with the best predictive performance. In addition, SHapleyAdditive exPlanation (SHAP) was used to interpret the decision-making ability of the selected models and an online web calculator was constructed to facilitate the use of the models (Hippisley-Cox et al., 2009). R software (version 4.3.2) was used for all statistical analyses, model construction and validation in this study.

## 3 Results

**Figure 1:**
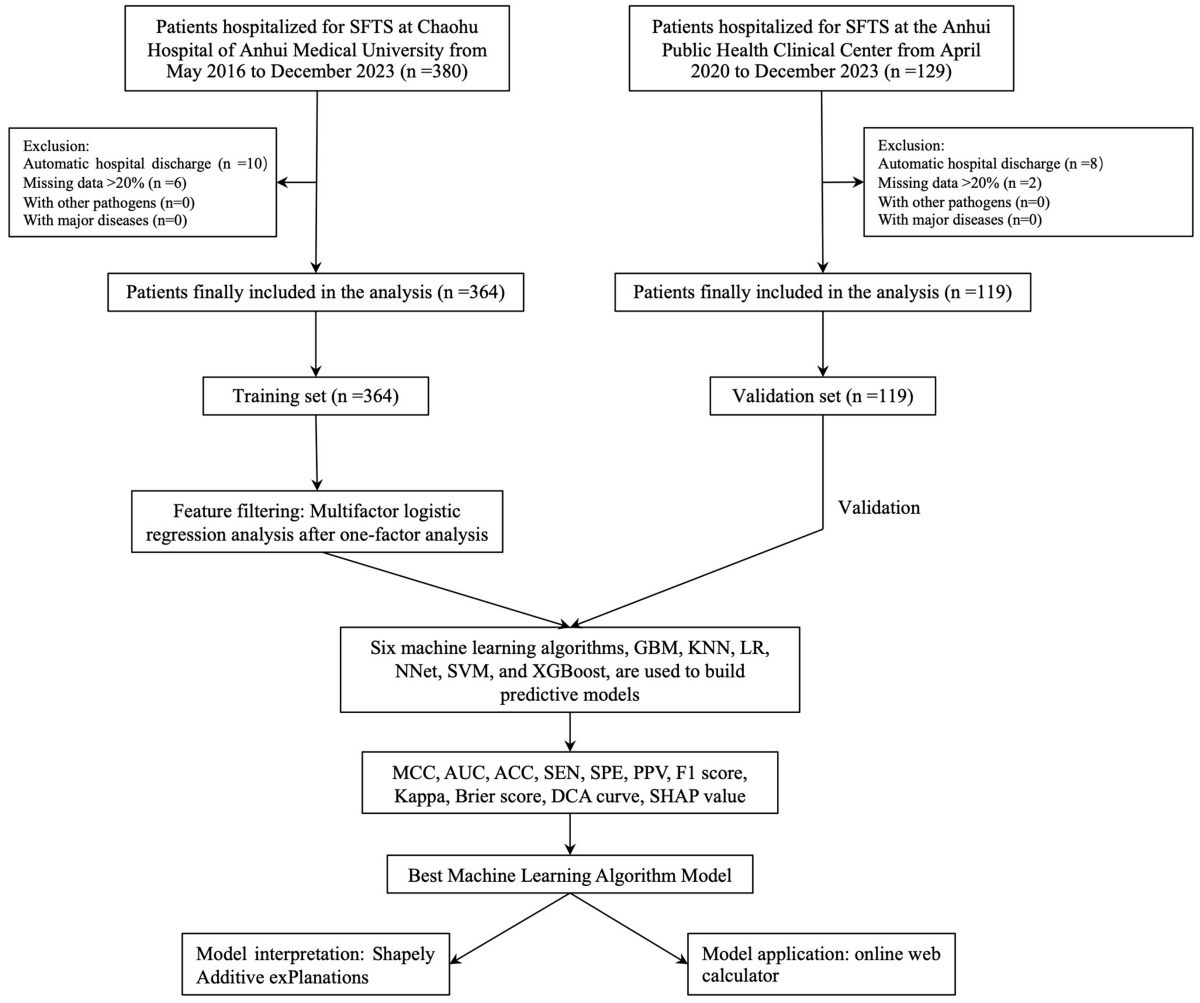
Flowchart of data screening and analysis.

After screening based on inclusion and exclusion criteria, a total of 483 participants were finally enrolled and 96 (19.88%) patients died due to SFTS. In Chaohu Hospital of Anhui Medical University, 364 patients were included in the study, of which 76 (20.88%) died, and in Anhui Public Health Center, 119 patients were included in the study, of which 20 (16.81%) died. The whole process of screening and analysis is shown in the flow chart (Figure 1). We extracted 46 variables from each patient, and the characterization of the missing data showed that the missing percentage of each variable was less than 15% in the training set and less than 12% in the validation set (S1). Missing data were interpolated using 50 iterations of the 5-fold interpolation technique. By comparing the density maps of each interpolation with the density maps of the original data, finally, we chose the value of the 1st interpolation as the final interpolation value for the training set and the value of the 4th interpolation as the final interpolation value for the validation set (S1).

The median age of the patients in the training and validation sets was 70 years (interquartile range [IQR], 59–76) and 70 years (IQR, 58.5–73.5), respectively; 212 (58.24%) and 61 (51.26%) patients were female, and the median time from onset of disease to admission was 4 days (IQR, 3–6.25) and 5 days (IQR, 3–6.5), respectively. The probability of patients having hypertension, CHD, diabetes, and CI in the training set was 14.01% (51 patients), 1.92% (7 patients), 6.59% (24 patients), and 4.40% (16 patients), respectively; while in the validation set it was 31.09% (37 patients), 4.20% (5 patients), 8.40% (10 patients), and 9.24% (11 patients), respectively. The percentages of clinical symptoms of myalgia, fatigue, nausea, emesis, diarrhea, AP, cough, dyspnea, and DOC at the time of admission in the training set were 39.29% (143 cases), 60.44% (220 cases), 37.64% (137 cases), 26.37% (96 cases), 35.99% (131 cases), 8.52% (31 cases), 18.96% (69 cases), 1.65% (6 cases) and 20.60% (75 cases); while the validation set was 36.13% (43 cases), 83.19% (99 cases), 31.93% (38 cases), 28.57% (34 cases), 47.06% (56 cases), 7.56% (9 cases), 19.33% (23 cases), 5.04% (6 cases), and 15.13% (18 cases). The patient characteristics of the training set are shown in Table 1.

**Table 1:** Characteristics of patients in the training set.

Variables	Total (*n* = 364)	0 (*n* = 288)	1 (*n* = 76)	*p*
Female	212 (58)	173 (60)	39 (51)	0.213
Age, Median (Q1, Q3)	70 (59, 76)	68 (57, 74)	75 (69.75, 79)	<0.001
DFOTA, Median (Q1, Q3)	4 (3, 6.25)	4 (3, 7)	4 (2, 5)	0.025
Hypertension, *n* (%)	51 (14)	31 (11)	20 (26)	0.001
CHD, *n* (%)	7 (2)	5 (2)	2 (3)	0.639
Diabetes, *n* (%)	24 (7)	16 (6)	8 (11)	0.196
CI, *n* (%)	16 (4)	6 (2)	10 (13)	<0.001
Temperature, Median (Q1, Q3)	37.6 (36.6, 38.4)	37.6 (36.6, 38.4)	37.6 (36.7, 38.23)	0.664
PR, Median (Q1, Q3)	82.5 (74, 93)	82 (73, 92)	85.5 (76, 100)	0.018
RR, Median (Q1, Q3)	20 (19, 21)	20 (19, 21)	20 (19, 21.25)	0.246
SBP, Median (Q1, Q3)	112.5 (100, 125.25)	112.5 (100, 125)	113.5 (99, 129)	0.634
DBP, Median (Q1, Q3)	70 (63, 78)	70 (63, 78)	72 (63.75, 80)	0.329
Myalgia, *n* (%)	143 (39)	113 (39)	30 (39)	1
Fatigue, *n* (%)	220 (60)	175 (61)	45 (59)	0.909
Nausea, *n* (%)	137 (38)	111 (39)	26 (34)	0.575
Emesis, *n* (%)	96 (26)	80 (28)	16 (21)	0.3
Diarrhea, *n* (%)	131 (36)	97 (34)	34 (45)	0.099
AP, *n* (%)	31 (9)	23 (8)	8 (11)	0.635
Cough, *n* (%)	69 (19)	53 (18)	16 (21)	0.719
Dyspnea, *n* (%)	6 (2)	5 (2)	1 (1)	1
DOC, *n* (%)	75 (21)	47 (16)	28 (37)	<0.001
Lymphadenopathy, *n* (%)	141 (39)	115 (40)	26 (34)	0.436
Hepatosplenomegaly, *n* (%)	1 (0)	1 (0)	0 (0)	1
WBC, Median (Q1, Q3)	2.07 (1.46, 3.19)	2.06 (1.44, 3.23)	2.16 (1.56, 3.02)	0.821
N, Median (Q1, Q3)	1.29 (0.82, 2.13)	1.21 (0.79, 2.13)	1.56 (1.06, 2.13)	0.057
L, Median (Q1, Q3)	0.54 (0.38, 0.79)	0.56 (0.39, 0.83)	0.47 (0.31, 0.66)	0.009
M, Median (Q1, Q3)	0.12 (0.08, 0.21)	0.13 (0.08, 0.23)	0.09 (0.06, 0.17)	0.004
HB, Median (Q1, Q3)	123 (112, 135.25)	123 (112, 135)	124.5 (110.75, 139.25)	0.331
PLT, Median (Q1, Q3)	56.5 (40, 74)	60 (45, 76)	46.5 (33.25, 60.75)	<0.001
ALT, Median (Q1, Q3)	53.5 (34, 93.75)	52 (33, 86.25)	55.5 (37.75, 113)	0.203
AST, Median (Q1, Q3)	132.5 (74, 261)	121 (71.75, 232)	171 (89.5, 432)	0.002
ALB, Median (Q1, Q3)	34.9 (31.5, 38)	35.1 (31.87, 38)	33.65 (31.08, 37.92)	0.189
GLO, Median (Q1, Q3)	28 (24.6, 31.7)	27.6 (24.5, 31.52)	29.25 (25.17, 32.12)	0.202
K^+^, Mean ± SD	3.68 ± 0.5	3.64 ± 0.48	3.86 ± 0.53	0.002
Ca^2+^, Median (Q1, Q3)	1.92 (1.85, 2.01)	1.94 (1.86, 2.02)	1.88 (1.81, 1.98)	<0.001
GLU, Median (Q1, Q3)	6.35 (5.5, 7.9)	6.3 (5.5, 7.4)	6.85 (5.9, 9.43)	0.003
BUN, Median (Q1, Q3)	6.6 (4.88, 9.4)	6.1 (4.6, 8.3)	9 (6.12, 12.25)	<0.001
CRE, Median (Q1, Q3)	76 (62, 100)	73 (61, 91)	101.5 (75.75, 140.5)	<0.001
LDH, Median (Q1, Q3)	552 (365.75, 875)	503 (357.25, 809)	648 (409.5, 1364.5)	0.008
CK, Median (Q1, Q3)	360 (176.5, 947.25)	339.5 (162.75, 807)	505.5 (213.5, 1375.25)	0.01
CK.MB, Median (Q1, Q3)	13.9 (5, 25.4)	13.04 (4, 23.22)	18.9 (8, 50.17)	0.001
PT, Median (Q1, Q3)	12.1 (11.1, 12.7)	11.9 (11, 12.7)	12.4 (11.78, 13.12)	<0.001
APTT, Median (Q1, Q3)	43.4 (37.8, 51.6)	42.4 (37.58, 49.7)	47.85 (41.27, 58.45)	<0.001
FIB, Median (Q1, Q3)	2.54 (2.2, 2.9)	2.57 (2.23, 2.9)	2.44 (2.17, 2.85)	0.256
TT, Median (Q1, Q3)	22 (19.4, 26.52)	21.6 (19.28, 25.02)	23.65 (19.98, 34.82)	0.002
D.D, Median (Q1, Q3)	3.05 (1.55, 6.7)	2.56 (1.41, 5.43)	6.44 (3.05, 12.06)	<0.001

DFOTA, days from onset to admission; CHD, coronary heart disease; CI, cerebral infarction; PR, pulse rate; RR, respiration rate; SBP, systolic blood pressure; DBP, diastolic blood pressure; AP, abdominal pain; DOC, disturbance of consciousness; WBC, white blood cell count; PLT, platelet count; N, neutrophil count; L, lymphocyte count; M, monocyte count; HB, hemoglobin; ALT, alanine transaminase; AST, aspartate transaminase; ALB, albumin; GLO, globulin; K^+^, potassium ions; Ca^2+^, calcium ions; GLU, glucose; BUN, blood urea nitrogen; CRE, creatinine; LDH, lactate dehydrogenase; CK, creatine kinase; CK.MB, creatine kinase isoenzyme; PT, prothrombin time; APTT, activated partial thromboplastin time; FIB, fibrinogen; TT, thromboplastin time; D-D, D-dimer; Q1, the first quartile; Q3, the third quartile. 0: patients who survived, 1: patients who died.

**Figure 2:**
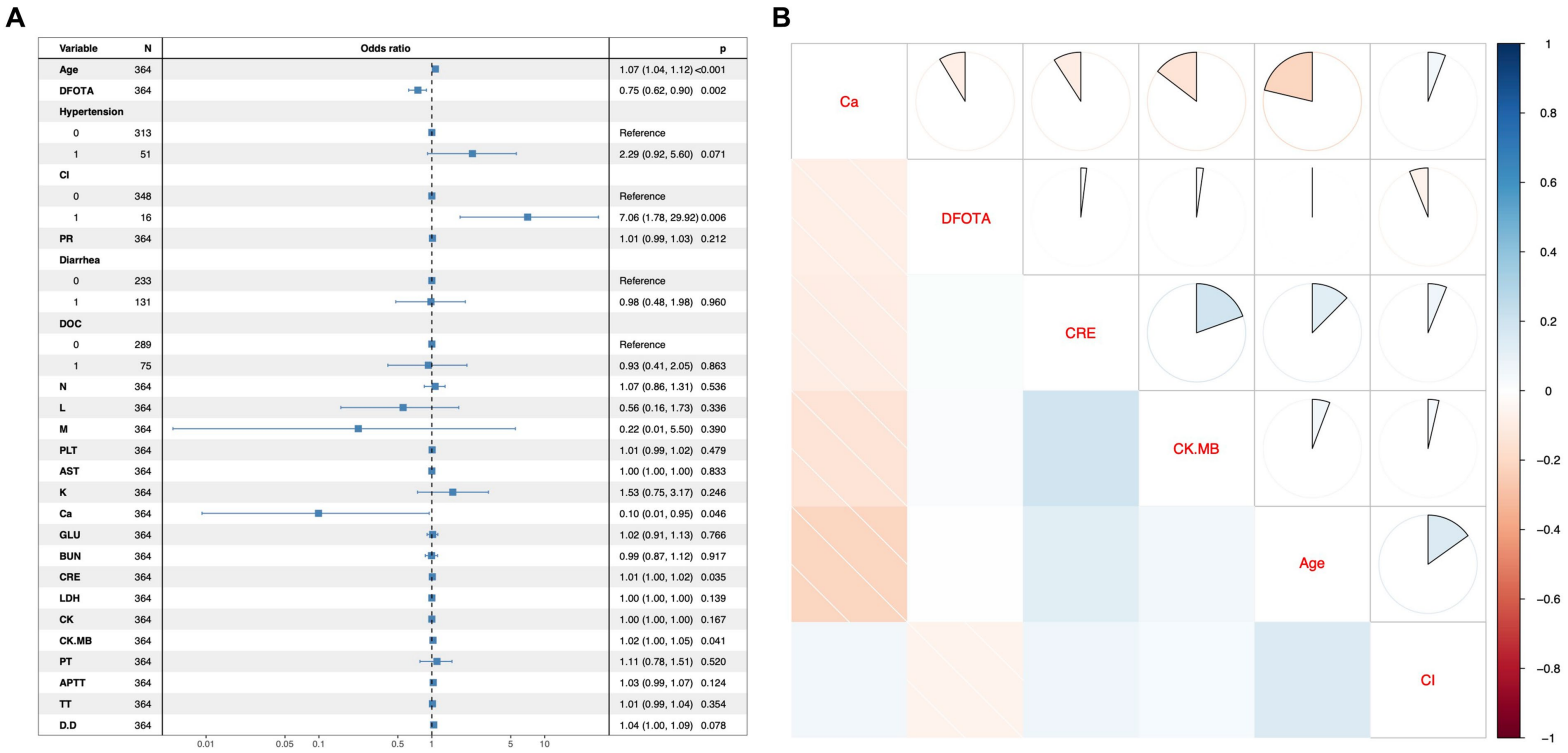
Multifactor regression forest plots and correlation analysis hotspots. **(A)** Forest plot for multifactor logistic regression model. The left column shows the variables included in the multifactor logistic regression model, with 0 and 1 representing “No” or “Yes” for the 2-categorical variables. The middle column shows the graphical representation of the Odds ratio. The right-hand column contains the odds ratio values and their 95% confidence intervals, and the rightmost *p*-value. An OR value with a 95% confidence interval that does not contain a 1 or a *p* value <0.05 indicates that the variable was statistically significant for the outcome in the multifactorial model. **(B)** Heatmap of correlation analysis between variables. The variables on the diagonal of this heatmap indicate that the rows and columns in which they are located are representative of that variable. Above the diagonal the correlation coefficients are shown as sectors, with a whole circle representing the absolute value of the correlation coefficient as 1. Below the diagonal, correlation coefficients are shown in shades of color, with the darker the color, the closer the absolute value of the correlation coefficient is to 1. Red is a negative correlation and blue is a positive correlation.

**Figure 3:**
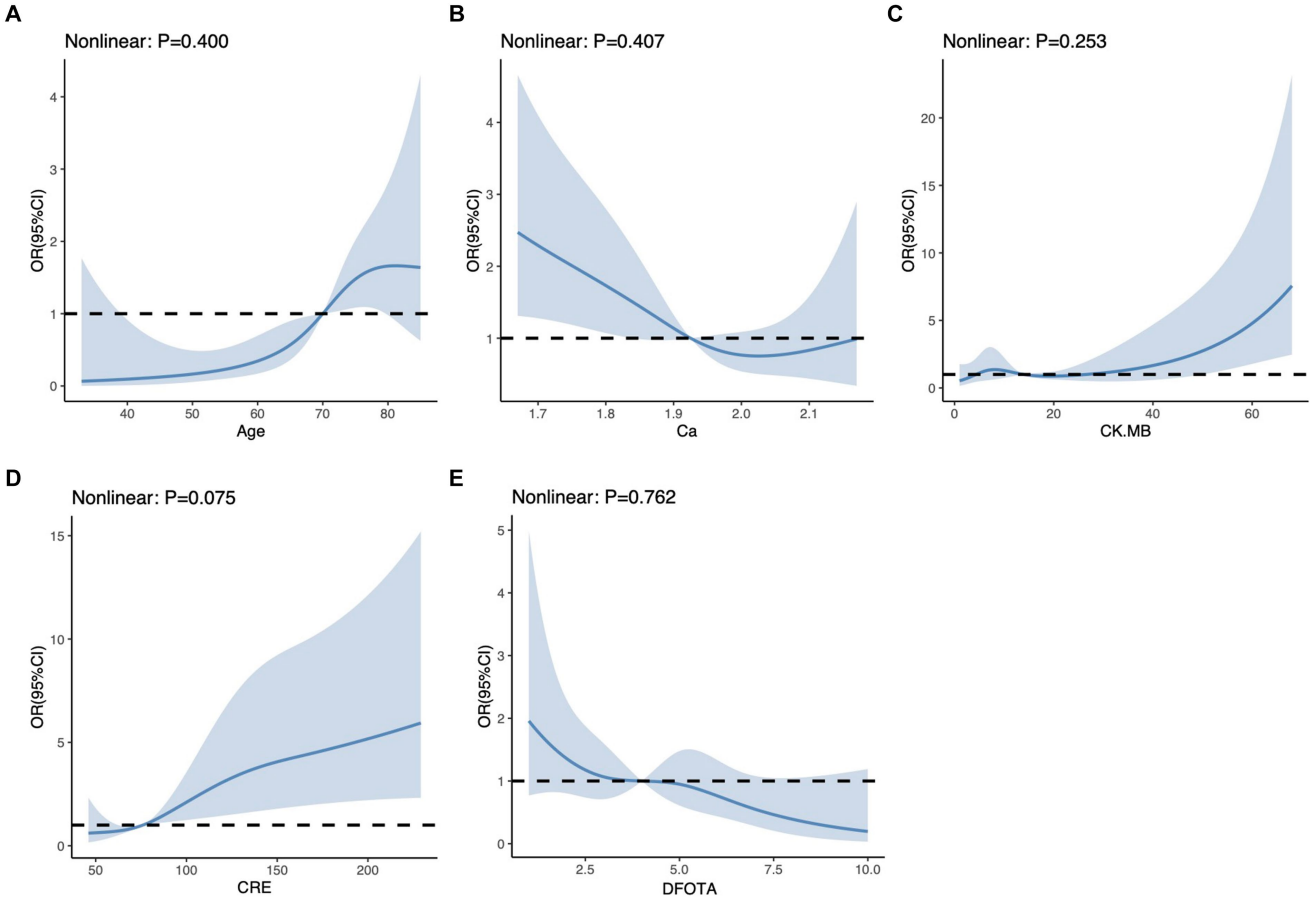
Restricted cubic spline for each continuous variable. **(A)** Restricted cubic spline for Age. **(B)** Restricted cubic spline for Ca. **(C)** Restricted cubic spline for CK.MB. **(D)** Restricted cubic spline for CRE. **(E)** Restricted cubic spline for DFOTA For each subplot, the horizontal coordinate represents the value of each variable and the vertical coordinate represents the ratio of the occurrence of the outcome event. The “nonlinear” in the upper left corner represents a test of nonlinearity between the variable and the outcome, which indicates a nonlinear relationship between the variable and the outcome when it is *p* < 0.05.

In this study, variables with *p* < 0.1 were used as potential risk factors for mortality outcomes, and the results of the analysis of variance indicated that Age, DFOTA, Hypertension, CI, PR, Diarrhea, DOC, N, L, M, PLT, AST, K^+^, Ca^2+^, GLU, BUN, CRE, LDH, CK, CK.MB, PT, APTT, TT, and D.D were potential risk factors for death after the occurrence of SFTS. After including these variables in a multifactorial logistic regression model, the results showed that Age [*p* < 0.001, OR(95%CI) = 1.07 (1.04, 1.12)], DFOTA [*p* = 0.002, OR(95%CI) = 0.75 (0.62, 0.90)], CI [*p* = 0.006, OR(95%CI) = 7.06 (1.78, 29.92)], Ca^2+^ [*p* = 0.046, OR(95%CI) = 0.1 (0.01, 0.95)], CRE [*p* = 0.035, OR(95%CI) = 1.01 (1.00, 1.02)], CK.MB [*p* = 0.041 OR(95%CI) = 1.02 (1.00, 1.05)] were risk factors for death after the occurrence of SFTS (Figure 2A). The heatmap of the correlation analysis (Figure 2B) shows that the correlations among the variables are less than 0.4, which indicates that there is no significant multicollinearity among the variables. The six variables, Age, DFOTA, CI, Ca^2+^, CRE, and CK.MB, do not interact with each other to cause problems such as instability of the model parameters and reduction of the model predictive ability, and thus all of them are used as the features of the machine learning model for constructing the model. In addition, we used RCS to explore the nonlinear relationship between variables and outcomes. Multivariate-adjusted RCS analysis showed that none of the five variables Age, DFOTA, Ca^2+^, CRE, and CK.MB had nonlinear relationships with death outcomes (Figure 3).

**Figure 4:**
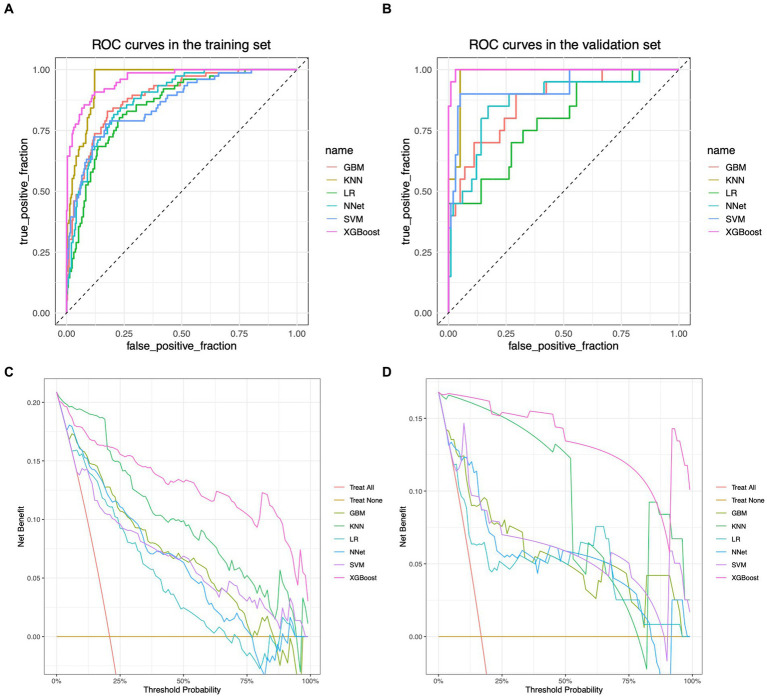
ROC curves and DCA curves for each model in the training and validation sets. **(A)** ROC curve in the training set. **(B)** ROC curve in the validation set. **(C)** DCA curve in the training set. **(D)** DCA curve in the validation set. The horizontal coordinates in graphs **(A,B)** are the false positive rates and the vertical coordinates are the true positive rates. Each curve of different color represents a different machine learning algorithm, and the machine learning algorithm corresponding to each color curve is labeled accordingly on the right side. The area enclosed by the curves and the horizontal and vertical coordinates can reflect the differentiation performance of the model, and the larger the area is, the better the differentiation performance of the model is. **(C,D)** are plotted with threshold probabilities in the horizontal and net clinical utility in the vertical. There are two straight lines in the graphs as reference lines, namely, the reference line where all samples are predicted to be positive (i.e., “all interventions”) and the reference line where all samples are predicted to be negative (i.e., “no interventions”), which are color-coded on the right side of the image. Each color curve corresponds to a corresponding machine learning algorithm, and the corresponding color of the algorithm is also marked on the right side of the image. Only if the model’s corresponding curve is above these two reference lines does the model achieve positive net clinical utility, with higher curves indicating greater clinical benefit from the predictions provided by the model at the corresponding thresholds.

**Figure 5:**
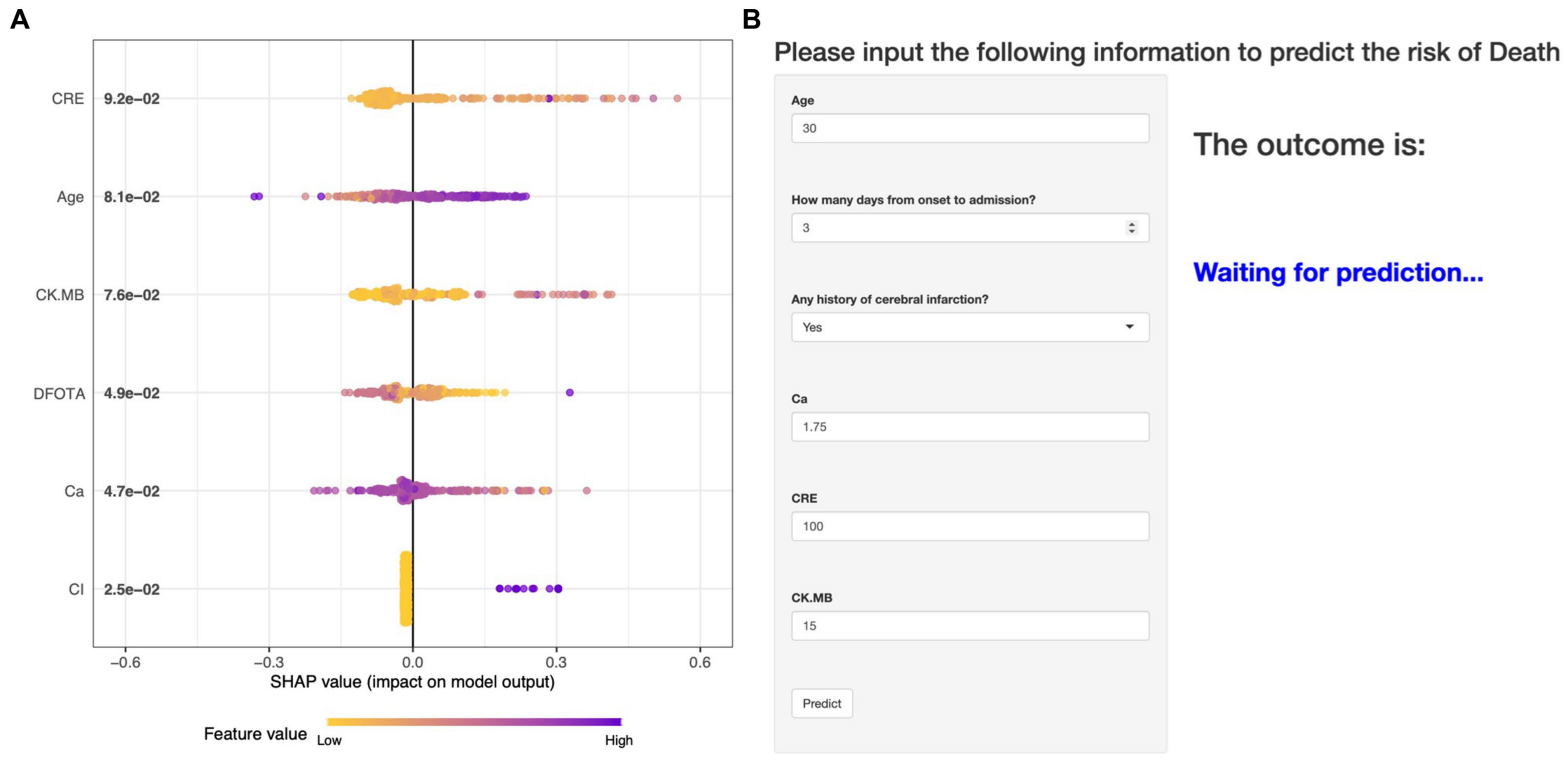
An online web calculator based on the XGBoost model and a summary plot of the SHAP values for the model. **(A)** Summary plot of SHAP values. The vertical coordinates are sorted in descending order of importance of the variables, with the higher up the scale the more important the variable is to the model. For horizontal positions, the “SHAP value” indicates whether the impact of the value is associated with a higher or lower predicted value. The color of each SHAP value point indicates whether the observation is high (purple) or low (yellow). **(B)** An online web calculator based on the XGBoost model.

The performance of the models constructed by each algorithm was determined by ten ten-fold cross-validation. Figures 4A,B show the ROC performance on the training and validation sets using the six ML methods, GBM, KNN, LR, NNet, SVM, XGBoost, respectively and the AUC values were calculated based on the ROC curves (Table 2). The AUC values (95% CI) of GBM, KNN, LR, NNet, SVM, XGBoost in the training set (Figure 4A) are 0.887 (0.848, 0.927), 0.957 (0.939, 0.975), 0.85 (0.805, 0.895), 0.88 (0.842, 0.919), 0.86 (0.812, 0.908), and 0.962 (0.941, 0.982), respectively; AUC values (95% CI) in the validation set (Figure 4B) were 0.87 (0.785, 0.955), 0.978 (0.957, 1), 0.788 (0.674, 0.903), 0.871 (0.779, 0.963), 0.93 (0.858, 1), and 0.997 (0.993, 1), respectively. Table 2 shows the detailed performance results on the training and validation sets using these six ML methods. It includes accuracy, recall, specificity, precision, Kappa value, MCC value, F1 score, and brier score. The MCC values of GBM, KNN, LR, NNet, SVM, XGBoost in the training set are: 0.571, 0.775, 0.490, 0.527, 0.539, 0.707; and in the validation set are: 0.464, 0.870, 0.636, 0.561, 0.803, 0.919. The F1 score of GBM, KNN, LR, NNet, SVM, XGBoost in the training set are: 0.663, 0.813, 0.601, 0.629, 0.641, 0.766; in the validation set are: 0.537, 0.889, 0.621, 0.630, 0.837, 0.930. The brier score of GBM, KNN, LR, NNet, SVM, and XGBoost in the training set are: 0.104, 0.081, 0.121, 0.109, 0.113, and 0.063; and in the validation set are: 0.092, 0.059, 0.105, 0.093, 0.129, and 0.034, respectively. Both in the training set (Figure 4C) and the validation set (Figure 4D), the DCA curves are able to lie above the none line and the all line in a wide range of thresholds across the models, where the models have clinical utility. Combining the various model evaluation metrics, XGBoost showed the best discrimination among all six ML models. In addition, a summary plot of SHAP values (Figure 5A) was used to interpret the XGBoost model results, which showed that the importance to the model was in the order of CRE, Age, and CK.MB. Finally, we also constructed an online web calculator for the XGBoost-based model that has the best performance for ease of use (Figure 5B, https://qionghan1999.shinyapps.io/SFTS/).

**Table 2:** Evaluation metrics of the models constructed by each algorithm.

		AUC	ACC	Recall	SPE	Precision	KAPPA	MCC	F1-score	Brier score
Train	GBM	0.887	0.824	0.829	0.823	0.553	0.551	0.571	0.663	0.104
	KNN	0.957	0.904	1	0.878	0.685	0.751	0.775	0.813	0.081
	LR	0.85	0.777	0.803	0.771	0.48	0.46	0.490	0.601	0.121
	NNet	0.88	0.799	0.816	0.795	0.512	0.502	0.527	0.629	0.109
	SVM	0.86	0.819	0.776	0.83	0.546	0.525	0.539	0.641	0.113
	XGBoost	0.962	0.885	0.908	0.878	0.663	0.692	0.707	0.766	0.063
Valid	GBM	0.87	0.739	0.9	0.707	0.383	0.395	0.464	0.537	0.092
	KNN	0.978	0.958	1	0.949	0.8	0.863	0.870	0.889	0.059
	LR	0.788	0.908	0.45	1	1	0.577	0.636	0.621	0.105
	NNet	0.871	0.832	0.85	0.828	0.5	0.53	0.561	0.630	0.093
	SVM	0.93	0.941	0.9	0.949	0.783	0.802	0.803	0.837	0.129
	XGBoost	0.997	0.975	1	0.97	0.87	0.915	0.919	0.930	0.034

Train, training set; valid, validation set; ACC, accuracy; SPE, specificity; MCC, Matthews correlation coefficient.

## 4 Discussion

In this study, six variables Age, DFOTA, CI, Ca, CRE, and CK.MB were screened by using data from Chaohu Hospital of Anhui Medical University and Anhui Provincial Public Health Center based on the method of constructing a multifactorial regression model after variance analysis, and these six variables were included as features in the ML model. We used six ML methods, GBM, KNN, LR, NNet, SVM, and XGBoost, to construct a prediction model for early identification of the risk of death in SFTS patients. We plotted ROC curves and calculated AUC values based on them, combined accuracy, recall, specificity, precision, Kappa value, MCC value, F1 Score, Brier Score to comprehensively evaluate the model performance, and plotted DCA curves to evaluate the clinical benefit of the predictive model application. Among the six ML models considered, the XGBoost model has the best performance. The AUC values (95% CI) of the XGBoost model for the training set and validation set are 0.962 (0.941, 0.982), 0.997 (0.993, 1), respectively, representing a good efficiency of the predictive model. For the unbalanced dataset in this study, the MCC values of the XGBoost model also showed good results in the training set (0.707) and validation set (0.919). F1 score of the XGBoost model in the training and validation sets were 0.766 and 0.930, respectively, which reflected the higher accuracy and stability of the model. In terms of the calibration of the model, the Brier score is 0.063 and 0.034 in the training set and validation set, respectively, which makes the model of the XGBoost method show the best performance. In addition, we use SHAP values and an online web calculator to solve the two major dilemmas of “interpretability” and “usability” that are widely found in machine learning predictive modeling research.

In this study, six variables, Age, DFOTA, CI, Ca, CRE, and CK.MB, were found to be high risk factors for death in SFTS patients. Many current studies have identified age as a key risk factor for death in SFTS patients (Yang et al., 2023; Liang et al., 2023; Zu et al., 2022; Shin et al., 2015; Wang et al., 2020). According to our study, DFOTA is also associated with mortality in patients with SFTS. However, we need to view this result with caution. The course of a patient’s SFTS can be broadly categorized into a febrile phase (3–7 days), a critical phase (7–13 days), and a recovery phase (11–17 days) (Gai et al., 2012). This also means that the further the patient is admitted to the hospital, the more likely it is that the patient is less ill. Future separate studies of patients with different staging are warranted. A novel finding of this study is that CI is a useful predictor of mortality in patients with SFTS. CI has previously been shown to severely affect disease prognosis in a number of studies (Hasegawa et al., 2014; Wasay et al., 2018; Djaharuddin et al., 2021). There are no corresponding studies examining the relationship between SFTS and CI. Therefore, further relevant studies are needed to confirm this risk in the future. The results of multifactorial logistic regression suggest that low calcium is a high risk factor for death in SFTS patients, which is consistent with the results of a retrospective study by Zheng et al. that included 327 SFTS patients (Zheng et al., 2023). Ca^2+^ is an important substance for maintaining normal physiological functions of the human body and is an indispensable ion for all physiological activities of the body. Its main physiological functions include the following: bone and tooth formation, nerve conduction, muscle contraction, cell signaling, blood clotting, maintenance of cell membrane stability and cell differentiation, etc. Therefore, the proper maintenance of Ca^2+^ levels is essential to maintain the normal functioning of all body systems. When Ca^2+^ is too low, a series of adverse reactions can be induced. Low levels of Ca^2+^ can lead to altered cell membrane potentials, which can affect the normal functioning of cardiac, nerve, and muscle cells, leading to cardiac insufficiency and neurological abnormalities, which may exacerbate the condition of SFTS patients. Ca^2+^ likewise plays an important role in the immune response as well, and low calcium levels may further exacerbate the inflammatory response, leading to worsening of the condition. Ca^2+^ is a key factor in the coagulation process, and low levels of Ca^2+^ can lead to coagulation dysfunction and an increased risk of bleeding. Patients with SFTS have inherent coagulation abnormalities, and hypocalcemia can further exacerbate the tendency to bleed and increase the risk of death. DBBV can cause damage to different organs, such as can invade the kidneys in the body (Guu et al., 2012). It is well known that CRE can be used to evaluate renal function. In this study, we found that CRE was an independent risk factor for death in SFTS patients, which is consistent with previous studies by Xu et al. (2018), Wang et al. (2017), and Liu et al. (2023). Similar to our findings, Gong et al. noted that SFTS patients who died exhibited elevated CK.MB early in the disease and that CK.MB was an independent early warning factor for death (Gong et al., 2021). In this study, the results of univariate analysis showed that both CK and CK-MB were potential risk factors for death in SFTS patients, while the results of multivariate regression analysis showed that only CK-MB was a risk factor for death in SFTS patients. Compared with CK, CK-MB has a higher sensitivity and specificity in determining myocardial injury, which is often associated with myocardial injury in patients with SFTS, and the level of CK-MB can more accurately reflect the myocardial injury of the patients. The results of the RCS analysis showed that the risk of death of the patients increased significantly with the elevation of the level of CK-MB. The results of RCS analysis showed that the risk of death increased significantly with the increase of CK-MB level, which also suggests that it is an important research direction for us to monitor the level of myocardial injury to determine the risk of death in SFTS patients. In addition, all previous risk factor studies have been based on linear relationships, which is not always the case in clinical settings between independent variables and outcomes. An important assumption of commonly used regression models is that the independent and dependent variables are linearly related. Therefore, nonlinear models are limited to fit with regression analysis. A better solution is to fit a nonlinear relationship between the independent and dependent variables. RCS is one of the most common methods for analyzing nonlinear relationships (Lee et al., 2018). In our study, RCS was used to explore nonlinear relationships. The correlation results in this study showed that none of the nonlinear relationships existed between Age, DFOTA, Ca^2+^, CRE, CK.MB, and mortality outcomes.

To the best of our knowledge, relevant studies are mainly in China at present. Prediction tools for predicting poor prognosis of death in SFTS have been developed by Qian et al. (2023), Wang et al. (2019), Zhang et al. (2023), and Li et al. (2023). The study by Qian et al. was a multicenter retrospective study that included 882 patients with SFTS and was characterized by a large sample size and different hospitals in different regions. A nomogram was constructed to predict the risk of death based on clinical characteristics and laboratory parameters, and the AUCs of the model were 0.898 and 0.890 in the training and validation cohorts, respectively. Instead, our research has developed ML models that are characterized by their ability to efficiently process big data and intricate patterns, giving the models better performance. The studies by Zhang et al. and Li et al. were single-center retrospective studies and lacked external validation. Compared with their studies, our study with a larger sample size and external validation can somewhat overcome the study bias and systematic errors and make the results more realistic. All of the above studies are based on the nomogram constructed by the traditional linear model. There are few studies based on ML constructing models about the prediction of mortality risk in SFTS patients, and only Zheng et al. built a model with the Reservoir Computing with Boosted Topology (RC-BT) method to predict the mortality of SFTS patients (Zheng et al., 2023). Ca^2+^, cholesterol, alcohol history, headache, field exposure, potassium, and dyspnea were identified as predictors of mortality in SFTS. Most of these indicators were retrospective variables and may be biased to some extent. Similar to our study, data were collected in a single center and validated with external data, and a more comprehensive performance evaluation of the model was performed: accuracy of 0.903, sensitivity of 0.913, specificity of 0.884, PPV of 0.809, NPV of 0.946, and AUC of 0.917. And in our study, the performance of the model was further improved relative to it. The clinical utility of the model was further evaluated and an online web calculator was constructed, which facilitates visualization of the clinical utility of the model and ease of use of the model.

Our study also has some limitations. First, the present study is a retrospective study, which may be subject to potential bias and confounding effects. Future prospective studies could largely avoid these biases. Second, although the sample size of this study could meet the minimum requirements for constructing a model (Moons et al., 2014), the number of positive events was small which could be statistically biased. Therefore, subsequent studies with large sample sizes to validate the model are essential. In addition, the populations in this study were all from eastern China, and the model should be viewed with caution when applied to populations in other regions. Further external validation of the model using population datasets from other regions and ethnicities is essential.

## 5 Conclusion

In this study, six ML models were constructed and evaluated by using SFTS patients from Chaohu Hospital of Anhui Medical University as the training set and SFTS patients from Anhui Provincial Public Health Clinical Center as the validation set. The final model based on the XGBoost method characterized by the six variables Age, DFOTA, CI, Ca^2+^, CRE, and CK.MB had the best performance. The model was further interpreted using SHAP with results suggesting that CRE, Age, and CK.MB are the top three important risk factors for death in patients with SFTS. Future prospective studies are needed to confirm this result. An online web calculator was also constructed to facilitate model application.

## Data Availability

The original contributions presented in the study are included in the article/Supplementary material, further inquiries can be directed to the corresponding author.
