# Supplementary material for: Machine learning for identifying risk of death in patients with severe fever with thrombocytopenia syndrome
Source: Front Microbiol. 2024 Sep 13;15:1458670. doi: 10.3389/fmicb.2024.1458670 (PMC11428110; doi:10.3389/fmicb.2024.1458670)
Supplement: Supplementary file 1 [file Data_Sheet_1.docx]

S1. Missing data analysis and interpolation plots.


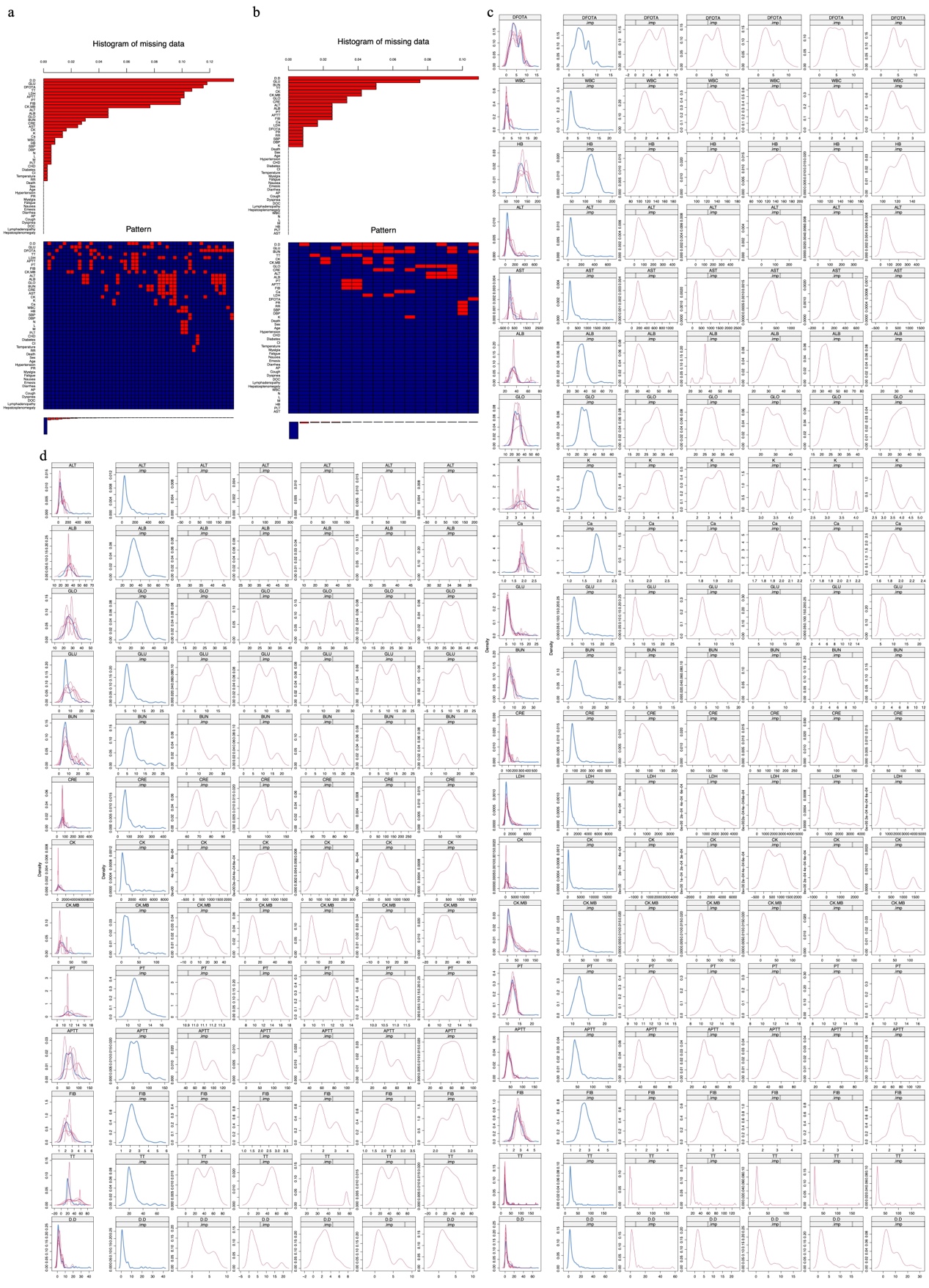


a: The upper histogram shows the percentage of missing data for each variable in the training set, with the horizontal coordinates indicating the percentage of that variable that is missing. The lower heatmap, where the red part indicates the missing values, shows the distribution of missing data for different variables in the training set.

b: The upper histogram shows the percentage of missing data for each variable in the validation set, with the horizontal coordinates indicating the percentage of that variable that is missing. The lower heatmap, where the red part indicates the missing values, shows the distribution of missing data for different variables in the validation set.

c: This figure shows the data feature density map of the training set data after 50 iterations of 5-fold interpolation technique processing. The blue curve represents the density map of the original data, and the red curve represents the density map of the data obtained from each interpolation, and the red curve from left to right corresponds to the 1st-5th interpolation in order. By comparing the density maps of the two colors, the effectiveness of the interpolation method in recovering the data distribution features of the training set can be evaluated.

d: This figure shows the data feature density map of the validation set data after 50 iterations of the 5-fold interpolation technique. The blue curve represents the density map of the original data, and the red curve represents the density map of the data obtained from each interpolation, and the red curve from left to right corresponds to the 1st-5th interpolation in order. By comparing the density maps of the two colors, the effectiveness of the interpolation method in recovering the data distribution features of the validation set can be evaluated.
